# Collective Self-Esteem and School Segregation in Chilean Secondary Students

**DOI:** 10.3389/fpsyg.2020.620011

**Published:** 2021-01-15

**Authors:** Olga Cuadros, Francisco Leal-Soto, Andrés Rubio, Benjamín Sánchez

**Affiliations:** ^1^Centro de Investigación para la Educación Inclusiva, Pontificia Universidad Católica de Valparaíso, Viña del Mar, Chile; ^2^Departamento de Ciencias Sociales, Universidad de Tarapacá, Iquique, Chile; ^3^Facultad de Economía y Negocios, Universidad Andres Bello, Santiago, Chile; ^4^Facultad de Psicología, Universidad Diego Portales, Santiago, Chile; ^5^Facultad de Educación y Ciencias Sociales, Universidad Andres Bello, Santiago, Chile

**Keywords:** collective self-esteem, segregation, psychometry, secondary education, belonging, social identity

## Abstract

Chile has established hybrid policies for the administrative distribution of its educational establishments, leading to significant gaps in educational results and school conditions between public, mixed, and private schools. As a result, there are high levels of segregation, and social and economic vulnerability that put public schools at a disadvantage, affecting their image and causing a constant decrease in enrollment. An abbreviated version of Luhtanen and Crocker’s (1992) collective self-esteem scale was adapted and validated for the Chilean educational context because of its usefulness in studying processes of social segregation and cultural coherence, seeking to identify student perception about the appreciation of school actions in the context of belonging and identification with schools, in order to compare between groups according to types of establishment and assess the effects of school conditions on the perception of students. A representative sample of Chilean secondary students between 9th and 12th grades participated (*n* = 3635, 52.8% women, average age 15.9 years, *SD* = 1.1). Descriptive analyses, comparison of means between groups, confirmatory factorial analyses, and multi-group analyses were conducted to test the adjustment and invariance of the unifactorial structure of a reduced version of four items. The results indicated that the scale satisfactorily complies with the proposed adjustment indexes, presents total invariance by gender and partial invariance by administrative dependence, and allows establishing statistically significant differences in the collective self-esteem, indicating a higher score for students in the private system, and a lower score for those in the public system. These results show the negative effects of high school segregation on students’ collective self-esteem, affecting the appreciation of personal, collective, and institutional activities and the sense of belonging. Although previous research has explored some of the effects of school segregation, the present study focuses on collective self-esteem, which is closely related to identity and belonging, and allows for further innovative research on school segregation. The scale is useful as an instrument for researching social conditions of student well-being, in regards to educational management.

## Introduction

The construct “collective self-esteem” describes the value that individuals make of themselves and of the activities that they carry out, based on the attitude and value manifested in the interactions with the group to which they belong and where these activities have been agreed upon. Based on this argument, Riia Luhtanen and Crocker (1992) designed a scale to measure this construct.

The use of this scale and its indicators has been extensive in social science research. In the educational context, studies have been developed on the identification of personal and social factors, helping to better understand the relationship that collective self-esteem has with the perception of worth, commitment, pride, satisfaction, confidence, and wellbeing, in terms of belonging to certain social groups.

A review of these studies shows that some of them place collective self-esteem as a protective factor in relation to the promotion of mental health in children, adolescents, and young people. An example of this has been research on students of migrant descent, with multi and intercultural identities considered an at-risk population regarding mental health, in which collective self-esteem shows direct effects on psychological, subjective, and socio-emotional well-being (Crocker et al., 1994; Kim and Omizo, 2005; Du et al., 2017; Cornejo et al., 2020). Other studies have been conducted on racial minority students whose educational opportunities are affected by intergenerational background (Thomas, 2017); others have focused on the interaction between family and school educational practices to show how collective self-esteem, as a cultural factor, promotes school engagement (Borrero and Yeh, 2020). Studies have also been published that show the effect of collective self-esteem on the self-construals responsible for psychological well-being in students at the higher education level (Yu et al., 2016). Other studies have been conducted to better understand social identity models based on intra-group practices, such as sport activities, physical education, and its propagation as a community activity in social networks, which strengthen group identity and cohesion, especially in highly competitive communities (Kim and Kim, 2019; Røset et al., 2020).

The development of collective self-esteem involves factors linked to an allocentric perspective of interaction between the psychological dimension and the cultural dimension of collectivism, with a key role in the development of group processes linked to the public and private dimension of activities, by establishing an interdependence between the normative beliefs that govern the social group and those available to the individual (Wu et al., 2011). This interdependence helps to partially explain phenomena of social interaction and coexistence within communities (Lim and Chang, 2009) or the levels of social cohesion and commitment within large groups (Khong et al., 2017; Schiefer and van der Noll, 2017), from a more sociological perspective. The present study is part of a broader research project of measurement of socio-emotional variables with a perspective that considers psychological and pedagogical development that can influence student trajectories, trying to account for the interaction between the world of the individual and the collective to which they belong. In this context, collective self-esteem was considered for the research as a variable to study the adherence, identification, and valuation of the actions carried out by the students according to their learning process, considering the cultural congruence of the student with respect to beliefs and attitudes promoted by belonging to educational establishments that are administratively and socioeconomically differentiated, recognizing that this linkage, in some cases, could explain phenomena of social segregation, prejudice, and social adjustment or adaptation (Bettencourt et al., 1999; Giang and Wittig, 2006; Rahim-Lateef, 2019; Jia et al., 2020), aspects that have become a topic of interest in Chile (Villalobos et al., 2020).

Previous research construct has established a relationship of collective self-esteem and the individual’s immediate environment at the individual and group levels, associated with factors of cultural or social identity, according to membership in certain communities. However, based on a review of the literature, there seem to be no studies that address collective self-esteem from an alternative approach that considers factors distant to the individual, such as management practices in the administration of the educational system based on public policy guidelines in education, which likewise have a direct effect on individuals and which, in the case of this study, determine differences in collective self-esteem based on the administrative segregation brought about by the different types of administration (public, private with state subsidy, and private) of educational establishments.

From this perspective, the goal of the study is to adapt and validate an abbreviated form of the scale of collective self-esteem in Chilean students to make it suitable for a specific use in the educational context, i.e., collective self-esteem in relation to belonging to a specific educational establishment. The study aims to collect the student perspective of integral individual-school assessment, looking for evidence that allows to identify relevant dimensions of commitment and interaction for the educational communities themselves, in order to support the design of action strategies for the improvement of educational quality, understood in an integral sense of psychological and social well-being, which transcends all participants in the educational communities and considers the institutional conditions under which they develop.

Chile is a country of significant social contrasts, magnified by the diversity of geopolitical conditions present in its long territory. On top of this geographical and cultural diversity, there are markedly segmented levels of social vulnerability reflected in the population and in schools, which show part of the complexity of the situation of extended poverty within the framework of a neoliberal social system, giving rise to accentuated processes of segregation and social exclusion (Álvarez-Ayuso and Cadena-Vargas, 2006). For this reason, Chile has one of the educational systems that are most subjected to processes of school segregation by socioeconomic level in Latin America. This type of segregation associated with socioeconomic level leads to large gaps in the social evaluation of the quality of education, according to the membership in institutions that vary according to the municipal agency (public), private-subsidized by the state (mixed) or private (Murillo and Duk, 2016; Santos and Elacqua, 2016; González, 2017). A fourth category, very few in number but whose characteristics deserve consideration, corresponds to establishments of public origin and financing that have been handed over to private institutions (foundations, in general) for administration (delegated). These categories correspond to the second variable considered for the present study, along with psychosocial vulnerability.

In today’s society, where there is a global interest in building and expanding communities and networks to ensure support and strengthen ties, individually perceived and socially concerted values regarding belonging and identification with certain groups and collectives become important (Rosenmann et al., 2016). In this process of identification, the interaction between trust and well-being perceived by individuals in relation to those perceived by other individuals who are part of the same collective stands out, as they identify themselves with the characteristics that define their groups of belonging within the same ecological context, which allows them to share elements that serve as a reference for integral development, both individual and collective (Olivos and Clayton, 2017).

This interaction between personal appreciation and the appreciation that others make of belonging to a group is what is called Collective Self-Esteem. In the collective self-esteem, the evaluation that people make of themselves, referring to their successes and failures in actions developed within a collective space, stand out, as well as the social comparisons with the evaluation that others make, considering their own internal parameters and the social judgment of the value of reference of these groups (Ramos-Oliveira, 2016). In this way, collective self-esteem comprises two major axes; on the one hand, the personal evaluation associated with processes of development and construction of identity that promote group membership, and the public evaluation of this membership, based on the collective aspect of self-esteem (Martiny and Rubin, 2016).

The self-esteem construct has been recognized for several decades since the theory of social identity (Luhtanen and Crocker, 1992). The social identity theory promotes the idea that the need for self-esteem motivates the members of a group to perform actions and maintain a perspective that holds up and protects the positivity of the group (Martiny and Rubin, 2016). For Luhtanen and Crocker (1992), this implies the construction of a collective self that seeks to strengthen internal interactions and identification within groups to maintain group cohesion and guarantee the long-term survival of the groups.

The collective self-esteem construct has been applied mainly to the field of social psychology, and has contributed to the study of prejudice, discrimination, and social segregation between communities, based on cultural and idiosyncratic factors (Kong, 2015). However, the construct involves the relationship between individual psychological aspects and social interaction that condenses, in a relevant way, the construction of the sense of social identity, considering aspects of cultural congruence (Dueñas and Gloria, 2017). This aspect of cultural congruence between individual and group allows the collective self-esteem construct to account for the levels of attachment, identification, and sense of belonging to culturally and socially situated groups; this is the case of educational contexts, considering the need to know the student’s perception and assessment with respect to their school contexts, demarcated by the configuration of communities that are territorially or culturally anchored to schools.

The school communities are configured as groups, characterized by the establishment of their own imprints, identities, and values defined institutionally in their pedagogical models, which they make public and spread to the external social environment to show their main attributes as ideal places for the formation of children and adolescents, hoping to obtain the approval, preference, and satisfaction of parents and guardians who decide to enroll the children in their care there (González et al., 2018). And, although the assessment of the schools made by the adults is important, it is even more important to consider the perspective of the students, which synthesizes the vision received from parents, other significant adults, peers, and other sources in the immediate environment, nuanced through the filter of their own experiences of acting and belonging in the schools, which account for the daily experience that provides key elements for their school careers. The collective self-esteem construct can offer an adjusted image of the students in relation to what they experience as part of the school collective, together with the appreciations that the students make of what others think of them and their school role.

## Materials and Methods

### Participants

A total of 3,635 high school students from years 9 to 12 of schooling (I to IV in the Chilean school system) from educational institutions selected through two-stage and stratified probability sampling, in the sixteen geopolitical regions of Chile, considering the four types of administrative units in the country: municipal (public), private subsidized by the state (mixed), private without state subsidy, and delegated administration. The schools were the first level units (selected through a simple randomization algorithm), and the final units were the classes selected within each school (which were selected through a Kish table). The Chilean Ministry of Education’s Enrollment Table 2017, a document that lists all the schools in the country, was used as the sampling frame. The average age of the participants was 15.9 years (*SD* = 1.1). Of the total sample, 1989 were women, corresponding to 52.8% in the sample, and 95% were born in Chile (the remaining 5% were distributed among South American nationalities, although there were also participants from North American, European, and Asian countries).

### Instruments

The present study was part of a bigger project regarding inclusive education and life trajectories of students in the Chilean school system, for which multiple variables and scales were used, including subjective well-being, self-regulation, and school climate, among many others. In the present study, the variables considered were: (a) Type of administrative dependence of the school establishments, and (b) Collective Self-esteem.

#### Collective Self-Esteem Scale (Luhtanen and Crocker, 1992)

This scale relates to the concept of collective identity from social identity theory (see Table 1). It starts from the idea that one of the social facets of individuals when they have membership within a collective is to perceive themselves as members of the group, sharing the same social category with all members (Luhtanen and Crocker, 1992). Following the ideas of Breckler and Greenwald (1986), Luhtanen and Crocker revisited three motivational facets of the self: public, private, and collective, to account for the personal evaluation that each person makes, based on how they see themselves in reference to the specific context of certain groups and their activities. Regardless of how each person perceives themselves in an isolated way, considering their attributes and capacities, belonging to the group introduces a factor that intervenes in the self-evaluation, contextualizing it based on the evaluation that is made of the group to which they belong and how they perceive that others evaluate the group.

**TABLE 1:** Items and dimensions of the complete CSES adapted for the school context.

#	Item	Dimension
1	I am a worthy representative of this school.	Membership
5*	I feel I don’t have much to offer to this school.	
9	I am a cooperative participant in this school.	
13*	I often feel I am a useless member of this school.	
2*	I often regret that I belong to this school.	Private self-esteem
6	In general, I am glad to be a member of this school.	
10*	Overall, I often feel that belonging to this school is not worthwhile.	
14	I feel good about being part of this school.	
3	In general, belonging to this school is considered good by others.	Public self-esteem
7*	Most people consider people from my school, on the average, to be more ineffective than others from other schools.	
11	In general, others respect the people from this school.	
15*	In general, others think that people from this school are unworthy.	
4*	Overall, the people from this school have very little to do with how I feel about myself.	Identity
8	The people from this school are a good reflection of who I am.	
12*	To my understanding, this school does not reflect the kind of person I am.	
16	In general, belonging to this school is an important part of my self-image.	

* Items reversed for scoring.

The construction of the original scale in 1990 considered four dimensions: membership esteem, which is the personal assessment of group membership (“I am a worthy member of the social groups to which I belong”); private collective self-esteem, which is the personal assessment of the value of the group itself (“I often regret belonging to some of my social groups,” an inverted item); public collective self-esteem, which is the value that other people make of the groups to which they belong (“In general, my social groups are considered good by others”); importance to identity, which is the importance given to group membership as an input for one’s self-concept (“The social groups to which I belong are not important in giving meaning to the type of person I am,” another inverted item).

The statistical factorial adaptation made by the test’s authors was based on a pilot application to 887 university students of different ethnic and cultural origins, which resulted in the final instrument consisting of 16 items selected as those with the best factorial load (between 0.54 and 0.83), with an explained variance of 60.7% and Cronbach’s alpha coefficients for each subscale and for the total scale above 0.83. The correlations between items reported for that pilot were 0.55 and higher (Luhtanen and Crocker, 1992).

This instrument was rephrased by the research team, to refer to the school, and the response scale was modified (see Table 2); thus, this version adapted to the school context is answered as a Likert scale with values from 1 (Strongly Disagree) to 5 (Strongly Agree).

**TABLE 2:** Items of the reduced CSES scale.

# Item	Content
1	I am a worthy representative of this school.
11	In general, others respect the people from this school.
14	I feel good about being part of this school.
16	In general, belonging to this school is an important part of my self-image.

A pilot administration was carried out on a small sample of secondary school students in the city of Iquique (Northern Chile), selected by availability (*n* = 414, school years 9 to 12, 54% women), to determine their comprehensibility and to carry out exploratory analyses that would allow the definition of items for an abbreviated version (see Table 3). Inter-item correlations in this sample were generally low, varying between *r* = 0.001 and *r* = 0.54. A EFA was executed to determine the emerging factor structure, and the result showed 5 factors with eigenvalues greater than 1, which does not correspond to the expected structures (one factor considering all items, or four related factors). The first of the factors covered half of the 40% variance explained by the set of items and presented several items with high loads, belonging to three of the four dimensions of the construct, only leaving one out of consideration. A new EFA was then executed, this time fixing the number of factors in four. The four factors explained 38% of variance, but the factorial loads did not allow assimilating the extracted factors with the dimensions of the construct, although in each one of them there was a marker —an item with high loading—that corresponded to each one of the dimensions, suggesting possible selectable items for an abbreviated version; but these items coincided only partially with those indicated in the previous analysis. A final EFA was executed forcing the extraction of a single factor, which explained 20% of the variance, but again one of the dimensions did not register any item with a load higher than 0.4. Finally, Cronbach’s alpha coefficient and item–total correlation was obtained for the set of items, resulting in an acceptable consistency (0.77) and low to moderate item–test correlations (between *r* = 0.1 and *r* = 0.58); but again, only three of the construct dimensions were represented among the items that showed correlations higher than *r* = 0.35 with the total score. Thus, a selection of items covering all four dimensions was not achieved, so it was decided to administer the set of items to the large sample, and to make the selection of items for the short version directly from that new sample.

**TABLE 3:** Sample size, averages, and standard deviations of the items of the scale, by administrative dependence.

*N* = 3635	Public *n* = *1330*		Mixed *n* = *1682*		Private *n* = *243*		Delegate *n* = *380*	
Item	*M*	*SD*	*M*	*SD*	*M*	*SD*	*M*	*SD*
1	3.00	1.12	3.05	1.11	3.20	1.18	3.02	1.08
11	3.22	0.99	3.35	1.00	3.47	0.99	3.21	1.00
14	3.27	1.00	3.33	1.02	3.43	1.00	3.31	1.01
16	3.05	1.04	3.07	1.02	2.97	1.08	3.02	1.02
Complete four-item scale	3.13	0.77	3.19	0.79	3.26	0.79	3.13	0.81

### Procedure

The scale was applied through self-report questionnaires during the 2017 school year, within the usual school schedule, as part of an instrument that included other scales in a larger study. The schools were contacted by phone with the purpose of inviting them to be part of the study. When the schools agreed to participate, they proceeded to deliver information about the study and schedule visits. In the event that they did not agree to participate, the next school on the list was contacted.

### Statistical Analysis

In order to evidence the psychometric properties and descriptive characteristics of the scale, descriptive analyses of correlations, tests of multivariate normality, confirmatory factor analysis (CFA), and factor invariance analysis incorporating absolute measures of fit, incremental measures of fit, and measures of comparative fit (Brown, 2015), as well as tests of differences in means between groups (one-factor ANOVA), and factorial ANOVA, were performed. This last analysis was carried out to test the capacity of the abbreviated instrument to capture differences between groups according to factors of institutional administrative dependence and the school vulnerability index (IVE), which in Chile are strongly associated with stratification by socio-economic level (NSE) and reported level of school vulnerability —calculated through the National System of Allocation with Equity (SINAE) and executed by JUNAEB (National Board of School Aid and Scholarships)—to identify the level of socio-economic vulnerability of groups of elementary and middle school students in public and mixed schools.

The statistical analyses were carried out using the support of the programs SPSS AMOS 22 (IBM Corp., 2013) and RStudio-IDE (Horton and Kleinman, 2015).

The factorial analysis (AFC) and invariance analysis were obtained using the weighted least squares estimator (WLS) to correct the underestimation of the factorial loads of items with asymmetric response frequencies, by establishing that the multivariate normality in the data is not met (Asún et al., 2016). Internal consistency coefficients were evaluated using McDonald’s Alpha and Omega indexes. Tests for gender and dependence invariance were performed evaluating configural invariance (same factor structure between groups, Model 1), metric invariance (same factor loads, Model 2), scalar invariance (same intercepts, Model 3), and same residuals within the multi-group confirmatory factor analysis (Model 4), sought to assess the magnitude of change in model fit rates at each level, following the guidance of Cheung and Rensvold (2002) regarding the application of the likelihood ratio test (differences in χ^2^ between nested models), and Δ-CFI < 0.01.

## Results

As a first step, the Kolmogorov-Smirnov test was performed for a sample, which indicated that the data did not follow a normal distribution (*p* < 0.05), and several descriptive analyses were performed for the entire 16-item instrument. Once the CFA was carried out following the original suggested 16-item structure distributed in four factors (membership esteem, private collective self-esteem, public collective self-esteem, and importance to identity), the result showed a poor fit in the selected sample of the school population: *N* = 3635, χ^2^ = 6745.627, df = 98, *p* = 0.00; IFC = 0.54, TLI = 0.44; RMSEA = 0.16; SRMR = 0.17 with dissimilar factor loads, from very low (0.03) to high (0.84).

From this poor adjustment of the model, a revision of literature about the construct was carried out (Branscombe and Wann, 1994; Ayestarán et al., 1996; Giang and Wittig, 2006), and in conjunction with the experts’ criterion, it was decided to try an abbreviated unifactorial model of four items, selecting the items of greater theoretical relevance reflected in the design arguments of the original scale by Luhtanen and Crocker (1992). The items were selected considering the highest probability of reflecting each of the dimensions contemplated by the construct, which are related to membership, perception of public, private and identity assessment of collective self-esteem. Likewise, that the items met conditions of higher factorial load in the complete model and moderate to low correlations was also considered.

Based on these orientations, items 1, 11, 14, and 16 were selected, which met all the theoretical and statistical conditions, regarding satisfactory factorial loads with the best behavior within the initial structure of each of the four factors of the base model (factorial loads 0.64, 0.77, 0.64, and 0.73, respectively).

The correlation between items was significant at low to moderate levels (coefficients between 0.34 and 0.50), with the highest value correlations between items 11 and 14 (*r* = 0.50) and items 14 and 16 (*r* = 0.50).

With this unifactorial structure composed of four items, the confirmatory analysis was carried out, for which this model shows a good fit: *N* = 3774, χ*2* = 5.394, df = 2, *p* = 0.06; IFC = 0.99, TLI = 0.98; *RMSEA* = 0.02; *SRMR* = 0.009, with significant factorial loads of 0.56, 0.82, 0.62, and 0.63, respectively, and McDonald’s reliability indexes ω = 0.75; Cronbach’s α = 0.75.

### Multigroup Invariance Analysis

Two models of invariance between groups were tested. The first was based on the gender variable (female, male and other) and the second on the administrative dependency variable (public, mixed, private and delegates). The relationship between the invariant models was analyzed by comparing the relative adjustment of each successively restricted model, following the assumptions mentioned by Cheung and Rensvold (2002) to identify the differences based on the indicators of χ*2* and IFC, with which the invariance assumption is accepted when p of χ^2^ > 0.05 and ΔCFI ≤ 0.01 (Tables 4, 5).

**TABLE 4:** Multigroupinvariance model adjustment indices by gender for the CSES scale.

		Absolute			Comparative				
Multigroup by gender	*df*	*X*^2^	RMSEA	SRMR	CFI	90% CI	Δχ^2^(Δdf)	*p value*	ΔCFI
Model 1 CSES configural	4	4.544	0.01	0.008	0.99	[0.000. 0.045]		0.33	
Model 2 CSES metric	7	12.267	0.02	0.01	0.99	[0.00. 0.047]	7.723(3)	0.09	0.00
Model 3 CSES scalar	10	15.796	0.02	0.02	0.99	[0.00. 0.041]	3.529 (3)	0.11	0.00
Model 4 CSES residual	14	22.222	0.02	0.02	0.98	[0.00. 0.038]	6.426(4)	0.07	–0.01

**TABLE 5:** Multigroup invariance model adjustment indices by administrative dependence for the CSES scale.

		Absolute			Comparative				
Multigroup by administrative dependence	*df*	*X*^2^	RMSEA	SRMR	CFI	90% CI	Δχ^2^(Δdf)	*p value*	ΔCFI
Model 1 CSES configural	8	8.005	0.001	0.009	1.00	[0.000. 0.039]		0.43	
Model 2 CSES metric	17	14.517	0.00	0.01	1.00	[0.00. 0.026]	6.512(9)	0.63	0.00
Model 3 CSES scalar	26	43.660	0.02	0.02	0.98	[0.012. 0.041]	29.143(9)	0.16	–0.02
Model 4 CSES residual	38	60.305	0.02	0.02	0.98	[0.12. 0.037]	16.645(12)	0.12	0.00

### Multi-Group Invariance Analysis, Based on Gender

The result indicates that the scale of collective self-esteem presents total invariance at all levels, giving an account of equality of factor structure, burdens, intercepts, and residues between groups of men and women.

### Analysis of Multi-Group Invariance, Based on the Administrative Dependence of the Establishment

The result of the multi-group analysis based on the administrative dependence factor of the educational establishments, indicates that the collective self-esteem scale presents partial invariance, showing equality of factor structure and factor loads among the established groups (public, mixed, private, and delegated), but does not present equality in intercepts and residues.

### Convergent Validation

The instrument included another series of scales to evaluate, among other variables, the satisfaction with life in students (Alfaro Inzunza et al., 2014), personal well-being (Bilbao Ramírez et al., 2016), and social well-being in school (Blanco and Díaz, 2005) taking up again the Keyes’ social well-being scale (Keyes, 1998). These scales were selected to carry out analyses of the associations with the average result of the collective self-esteem scale, given its compatibility and relevance in terms of the content measured, which refers, among other aspects, to feeling satisfied with the school and with the self-confidence it provides when participating in its activities, in addition to investigating the students’ perception of being an important part of the school community, the evaluation they receive from the people linked to the school, the closeness to these people linked to the school, the school as a source of well-being, the personal contribution that the students makes to the school, and the importance of this contribution, the school as a place of continuous improvement for the students, the school as a source of opportunities, the school as an institution that advances and improves, and the value of the school itself in relation to other schools, considering dimensions of social integration, social acceptance, social contribution, social actualization, and social coherence. As can be seen in the results of the correlations in Table 6, all the correlations are statistically significant, and are established in an expected positive direction.

**TABLE 6:** Correlations of the global score of the Collective Self-Esteem Scale and the Satisfaction with Life Scale for students, Personal Well-being, and Social Well-being.

	1	2	3	4
1. CSES Mean	1.00			
2. Satisfaction with life	0.26**	1.00		
3. Personal well-being	0.29**	0.84**	1.00	
4. Social well-being	0.27**	0.24**	0.26**	1.00

**p < 0.01.

### Results of Comparison of Means Between Groups According to Administrative Dependency Factors and Vulnerability Index

The first factor established was the administrative dependence of the educational establishment, and the second factor was the vulnerability index within the composition of the student population in each establishment.

The administrative unit was established at four levels (public, mixed, private, and delegated). The vulnerability index was established at three levels corresponding to low, medium, and high.

First, Levene’s test was established and the homogeneity of the variances was tested (*p* > 0.05). Differences between means were established with a *post hoc* Scheffé’s test using one-factor ANOVA, which resulted in significant differences in the administrative dependency factor [*F*(3,3631) = 3.04, *p* < 0.05], and differences in means (I-J) *p* < 0.05 were identified between municipal and non-subsidized private establishments. For the vulnerability index factor, the differences are also significant [*F*(2,3632) = 4.09, *p* < 0.0] identifying mean differences (I-J) *p* < 0.05 between the medium and high levels of vulnerability.

To control a possible interaction between both factors, a factorial ANOVA (2 × 4 × 3) was conducted with the two factors as independent variables associated with dependency and vulnerability index. The interaction between factors was not statistically significant, while the main effects of each factor (administrative dependency and vulnerability index) on the measurement of collective self-esteem were statistically significant: *F*(2,3634) = 3.173, *p* < 0.05; *F*(3,3634) = 2.641, *p* < 0.05 correspondingly.

**FIGURE 1:**
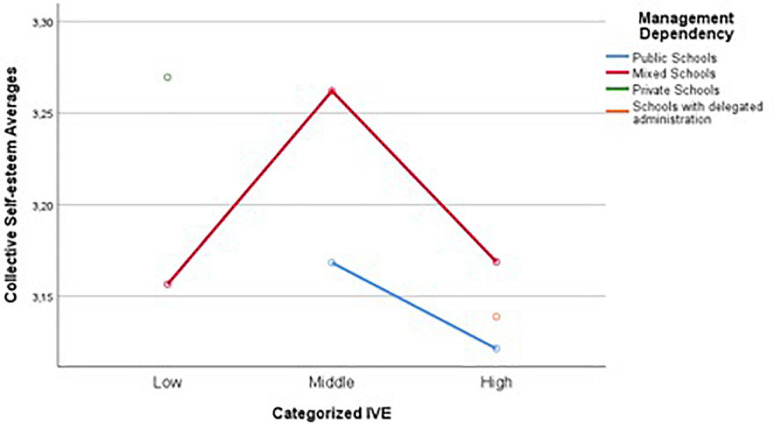
Main effects of the vulnerability index and the type of administrative dependency of the schools on the levels of collective self-esteem.

Figure 1 shows that public school students with higher vulnerability indexes have lower average scores in collective self-esteem, and individuals whose vulnerability indexes are low, have the highest average scores in collective self-esteem. Although the interaction of factors is not significant, the distribution of average scores in collective self-esteem in mixed schools is noteworthy, indicating that within this level of schools, those with a medium vulnerability index obtain better average scores in collective self-esteem.

## Discussion

The main purpose of this work has been to adapt and validate an abbreviated version of the collective self-esteem scale to make it a brief, easy to use, and graded instrument, suitable for a massive use in educational context to measure students’ perception of their own assessment and confidence and of their activities carried out according to their belonging to the school as a reference group. The analyses carried out allow us to establish the achievement of this psychometric objective.

The revision of the adjustment of the complete scale allowed identifying the items with better demonstrative capacity of the collective self-esteem construct, facilitating the selection of representative items of the four dimensions that originally integrate the scale, this time within a unifactorial structure. This unifactorial solution showed a good adjustment, allowing to configure a brief, understandable instrument, easy to apply, and answer by the students, which reflects in its content the main characteristics proposed for the collective self-esteem construct, attending to the ideas that underlie the construct regarding the personal appreciation associated to development processes and identity construction promoted by group membership, and on the other hand, the public appreciation of this membership, based on the collective nature of self-esteem (Martiny and Rubin, 2016).

Multigroup factor invariance analyses conducted on the basis of gender and administrative dependence of the establishments indicate that the scale meets measurement criteria without variation among these established groups. In the case of gender analysis, the invariance is complete, constraining the equality of factor structure, burdens, intercepts, and residues between the groups. In the case of multi-group analysis based on the administrative dependency factor of the establishment, the invariance between the groups determines the equality of the factor structure and the burdens between the different groups. The intercepts and residuals vary depending, most likely, on aspects that introduce specific variance attributable to cultural factors (Cheung and Rensvold, 2009), without altering the equality of measurement from the understanding of the items and their structure with respect to the collective self-esteem construct. In any case, the results of both multi-group analyses allow us to support the scale’s capacity to indicate reliable and stable measurements, with equal comprehension, through its application among different sample groups, based on these factors.

Convergent validity was examined and confirmed with three selected scales. The overall score of the collective self-esteem scale was positively correlated with the satisfaction, personal well-being, and social well-being scales, indicating that the content of the collective self-esteem scale points in a direction consistent and coherent with other instruments designed to establish psychological and social measurements in the school context.

The descriptive results made it possible to account for the variations in scores obtained, differentiated by type of establishment according to its administrative dependency, which was established according to four groups within the sample: public establishments with a municipal dependency, mixed, private establishments, and those delegated to private corporations. The results indicated lower average scores in the collective self-esteem of students belonging to public and delegated schools, reflecting, according to what the literature shows about school segregation processes in Chile, that there are large gaps in results between schools, according to a factor of socio-economic segregation and availability of institutional educational resources (Bellei, 2013). According to this, there is less confidence and appreciation in the activities carried out when belonging to this type of schools (public and delegated) in relation to mixed and private ones. The literature supports the idea that there is a strong influence of the processes of segregation in educational institutions according to their administrative dependence, with respect to the loss of confidence in the quality of public educational institutions and/or those that take in a large number of students in conditions of socio-economic vulnerability, in the context of the crisis in the quality of the public education system in the country (Bellei et al., 2010; Verger et al., 2016).

Around what has been called the “crisis of public education in Chile” there is a shared collective belief that these public educational establishments, which are mostly attended by the most vulnerable student population in the country, do not meet the standards of educational quality, as they are subject to the effects of the transfer of their administration to municipal entities without competence for educational administration (Bloque Social, 2006), a product of a reform of municipalization of the public education system carried out in 1986, during the period of the military dictatorship in Chile, while at the same time the boom in educational offer in private establishments was promoted. This introduced an asymmetric impulse in the educational system, generating an educational market offer that resulted in high levels of social and economic segregation, by promoting better educational conditions for those families with available resources and the ability to pay for private institutions, and leaving public education as the default option for those unable to pay the high costs associated with education in Chile (Assaél et al., 2015). The performative effects of this practice of managing the supply of education led to the precariousness of the public education system, since it did not have adequate educational management and administration processes, which began to be observed in a systematic decrease in enrollment at this level, and a decline in the results of evaluation of educational quality indicators (Madrid-Castro, 2020). As part of a negative cycle, these educational management problems were linked to problems of school coexistence, associated with high rates of socioeconomic vulnerability of the student population within the public system (Ligueño-Espinoza et al., 2018). As a result, the social perception of the public education system deteriorated to the point that it is considered an achievement to make a social escalation by having the opportunity to leave the public education system to enter the private or mixed system, having through a strong bias of representation about the quality of education and the membership in this type of establishment (Canales et al., 2016). This same category of precariousness also includes delegated administration schools, which are also a figure inherited from the military dictatorship, in which some technical-professional schools were transferred for administration to “non-profit” foundations, but with a clear business and trade union objective (Zurita-Garrido, 2018). Under this sociological and socio-historical perspective of the impoverishment of the educational identity of the public system and the exaltation of the private system in Chile, the results of the distribution of averages obtained by the scale of collective self-esteem at each level of dependency make a lot of sense, the highest averages being those obtained by students belonging to the private and mixed systems, which validates the hypotheses of the studies of school management and development that are established with a focus on the segmentation and segregation that is generated among the different modalities of administrative dependence of educational establishments, and that echo in the processes of identification and development of the sense of belonging in students, as indicated by the results of the collective self-esteem scale.

The interaction between dependency factors and the vulnerability index did not show a statistically significant effect, which can be attributed, on the one hand, to the fact that both factors are usually strongly associated, and, on the other hand, to the lack of dispersion and variability in the vulnerability indexes at two of the four levels in the administrative dependency factor. That is, at the level corresponding to the private system, the vulnerability index reported for the entire level was homogeneously low, while at the level corresponding to the delegated administration system, the vulnerability index was homogeneously high.

In this way, the analysis of the main effects within this model of group comparisons allows us to establish, as seen in Figure 1, that at the level of public establishments, students obtain a higher average result in collective self-esteem in the presence of a medium level of vulnerability (in the sample of public establishments there is no report of low levels of vulnerability), and as the student vulnerability index increases there is a decrease in the average obtained in collective self-esteem.

In the case of mixed schools, the results indicate that in the presence of low vulnerability indexes, the results obtained in the collective self-esteem of students are lower compared to the case of medium and high vulnerability indexes. The highest scores of collective self-esteem are found in students belonging to schools with a medium vulnerability index. In this type of mixed schools, in the cases corresponding to a high vulnerability index, the student population presents more homogeneous characteristics in socioeconomic terms.

When there is a low or high vulnerability index, the school populations tend to be homogeneous; in the case of the low vulnerability index, this could mean the condensation of students with no major socioeconomic needs, who by sharing some socioeconomic privileges, from the beginning and throughout their school careers, would not have the need to reinforce their collective identity, since from the outset they have the recognition of their individual strengths and confidence in the development of their activities within their educational establishments, which usually have an excellent reputation and educational results. In the case of the high vulnerability index, the homogeneity of the student population also plays against the attention given to membership and belonging to the educational establishment within the processes of collective self-esteem, considering that in this population there are greater problems of coexistence and satisfaction of basic needs, which ends up directing resources to individual needs, interfering with the possibility of generating processes of cultural adaptation and school identity in these establishments (Ligueño-Espinoza et al., 2018). The cases corresponding to the medium vulnerability index of the particular schools subsidized in the results share the school membership among students with different types of needs and socioeconomic conditions, which would tend to decrease school segregation, and which, according to studies on the benefits of more inclusive and less segregated schools, favors interactions that generate common social identification points to develop processes of school balance and adaptation (Ortiz, 2015, 2018).

Based on the results found, which show the influence that the presence of mixed populations has on their vulnerability indexes (indicated by the medium vulnerability indexes) in the increase of collective self-esteem levels, mainly in public and mixed schools, the authors consider school integration by socioeconomic levels as a positive and protective factor for the development of a collective sense of self-esteem, appealing to the promotion of a real perspective of diversity and inclusion in educational establishments. However, it would also be necessary to study in greater depth the processes of building value and institutional seals that are embodied in institutional educational projects (PEI) and that respond to the construction of processes of collective identification, which could directly influence the appreciation of groups and active membership in them, considering that in Chile the public, mixed, private and delegated administration systems coexist; in addition to regulations that establish that each establishment, regardless of its administrative dependence, must embody in its institutional educational project the values and cultural seals that distinguish them. In theory, this means having a wide variety of educational projects to which parents and guardians can turn in order to choose the educational institution that best suits their needs and with which the children and adolescents can be represented. In practice, this is not the case because, in the first instance, the selection of the institution for the children is not based on the educational seal that the institution presents, but rather on conditions of geographic location, school climate, costs, and method of selection (Villalobos and Salazar, 2014). Second, although all schools have IEPs, the institutional identity in many of them is neutral (mainly public), based on homogeneous values, commitments, and standards, which do not manage to establish a point of differentiation, which in some cases is evident because public schools are usually recognized by letters and numbers, losing the identity of a particular name (Alvarado Agurto, 2015). Since this aspect was not included in this study, it is established as a research variable in future projects on the topic.

Based on the results described and discussed, this study provides a useful, psychometrically sound instrument to identify the assessment processes that support collective self-esteem in the context of the subjective evaluation of school group membership in secondary students. Furthermore, it constitutes a valuable instrument with the capacity to identify variations in collective self-esteem, based on the social and contextual conditions of the highly segregated environments that characterize certain educational communities, especially in a country like Chile. Thus, an important contribution of this study, is that it provides evidence of psychometric properties of the collective self-esteem scale, considering the students’ membership in a specific educational establishment, with particular administrative characteristics, which is a contribution to the study of well-being and group influence phenomena, of interest to both the social sciences and education.

The main contributions that the study of collective self-esteem has made to the social and educational field have been to identify biases within groups, linked to the interaction between personal self-esteem, collective self-esteem (as a function of large groups) and even relational self-esteem (established on the basis of networks built with those close to them), and that account for the estimates that individuals make when they value and qualify their activities and the personal value they place on themselves when carrying them out, depending on the social group with which they identify themselves socially and culturally, and the one in which they carry out these activities (which do not always coincide). Thus, the study of collective self-esteem has allowed the identification of diverse problems and interactions at the level of interpersonal relationships, depending on the social and cultural identity of the people within the groups to which they are emotionally, culturally, and valuably anchored by a process of subjective and historical construction. In this case, the study has made visible a problematic reality of identification and sense of social belonging within educational communities. The decision to belong to a particular educational institution, preferably by the family and the student, is free and voluntary in the case of socio-economically privileged communities, but in the case of communities with lower socio-economic levels this decision is not. It ends up being a selection by default, arbitrarily defined according to socio-educational categorizations and management that does not respond to a natural disposition of individuals to interact among themselves freely and voluntarily, according to their personal characteristics, their beliefs or their culturally determined and diverse practices, but rather due to their socio-economic limitations, imposed through school segmentation, in territories that are socio-economically fragmented by interests that respond to the exercise of capitalism applied to education.

In this way, the use of the collective self-esteem construct within this study allows us to explain the mechanisms through which the student gives meaning to their activity within the framework of belonging to a determined group, based on the difference in the type of establishment in which they study. This difference is determined by the public policies of educational administration and the possibilities of school choice based on the socioeconomic level of individuals and communities, over which individuals have little or no influence in the case of the most vulnerable sectors (which generally correspond to the level of public education), but are important determinants of their sense of belonging and well-being, as processes based on social identification, facilitators of social incorporation and adaptation (Long et al., 1994; Jetten et al., 2002; Jensen, 2020). The contribution of this study to the understanding of how variables of perception and development of the individual’s social identity are sustained in conditions of management of their educational context is important, considering the enormous social and cultural influence, as well as values, that membership in a particular school educational community involves. This allows for a better perception of the connections between the disposition of public policies applied in education and the subjective results of students, given the possible implications this may have in the development of school trajectories (Eccles et al., 2006; Kim and Kim, 2019).

An interesting perspective to develop future studies from these results points to the incorporation of other variables of a contextual nature, which help to complement the information that describes the scale of collective self-esteem. As indicated in the introduction and the methodology, this study was considered an initial step in the analysis of the contextual variable collective self-esteem, incorporated along with other variables within a broader study, and whose main objective was to validate the scale in the Chilean school population and to report the main results measured in a diverse sample at the national level, hoping to be able to describe its effects based on the differentiation established from the administrative dependency, a critical issue in Chile and which represents a strength and contribution of the study considering that a representative sample of the target population was covered. Although the literature indicates that there are a number of other related variables based on broad constructs, or meta-constructs (Torres et al., 2018; Huguley et al., 2019), related for example to the allocentric perspective (Glaveanu, 2019) and welfare (Valcke et al., 2020) that account for the normative interaction between the subjective and the collective dimension with a social ontological character (Welch and Yates, 2018), they were not incorporated in this specific study, nor in the broader study of which it is a part, given that this was not the general objective established for the design of the project in general, as it considered the measurement of socio-emotional variables with a psychological and pedagogical development perspective, that influence student trajectories. The introduction of this type of variables would have widened the spectrum and objectives of study, exceeding therefore the initial vision closer to the educational field. However, the results obtained and reported in this manuscript open a perspective that deserves further exploration, so the introduction of these variables as part of a broader explanatory social model is a challenge to be addressed in the near future.

In that sense, the validation process of this scale in Chilean school population allows, precisely, to have a measurement tool that highlights the psychological and social effects that entails neoliberal practices of control and population segmentation linked to exclusion and prejudice associated to wealth/poverty conditions in the territories.

## Data Availability Statement

The raw data supporting the conclusion of this article will be made available by the authors, without undue reservation.

## Ethics Statement

The studies involving human participants were reviewed and approved by Comité de Bioética, Pontificia Universidad Católica de Valparaíso. Written informed consent to participate in this study was provided by the participants’ legal guardian/next of kin.

## Author Contributions

OC contributed to the conception and design, analysis, interpretation of data, drafted the work, revised it critically for important intellectual content, approved the final version of the manuscript to be published, and agreed to be accountable for all aspects of the work. FL-S contributed to the conception, analysis, and interpretation of data, drafted some parts of the work, revised it critically for important intellectual content, and approved the final version of the manuscript to be published. AR performed the measurements, sample design, aided in interpreting the results and worked on the manuscript, revised it critically, and approved the final version of the manuscript to be published. BS contributed to the acquisition of data, contributed to the analysis and interpretation of the results, drafted parts of the work, revised it critically, and approved the final version of the manuscript to be published. All authors contributed to the article and approved the submitted version.

## Conflict of Interest

The authors declare that the research was conducted in the absence of any commercial or financial relationships that could be construed as a potential conflict of interest.
